# The Department of Modern Nursing

**Published:** 1915-01-30

**Authors:** 


### THE DEPARTMENT OF MODERN NURSING.*

### VIII.—London Homœopathic Hospital.

It may be said at once that the distinctive medical aspect of the work carried on at the London Homœopathic Hospital does not in any degree differentiate the training given to the nurses from that which they would receive in an allopathic establishment. It is necessary to lay stress on this fact in case it should be imagined that nurses trained in a homœopathic institution are instilled with theories which might militate against their success in a broad field of work. So far as all nursing details are concerned, the treatment of patients is identical with that of every modern hospital. The hospital contains 163 beds, and the nursing staff consists of the matron, with assistant, housekeeper, night sister, seven sisters, fourteen charge and staff nurses, and forty probationers.

### First-Year Probationers.

The age-limit is from twenty-two to thirty-two, and accepted probationers agree to serve the hospital for four years. Candidates serve for two months on probation, receiving no salary unless appointed probationers. There is no preliminary school, and on their first entrance into the hospital the newcomers serve first in the ward kitchens and then as bathroom probationers, with a certain amount of ward work. During their first year they are attached alternately to medical and surgical wards, children, or out-patients—three months in each, and do no night work. They attend lectures on the Outlines of General Anatomy and on the Elements of Physiology. Their classroom is quite admirably supplied with all the apparatus likely to be useful in imparting these subjects, and there are few training schools which have so systematically grappled with the difficulties of making these studies lucid to their pupils. The equipment of the classrooms is equal to that furnished in high-class technical colleges, and the fine models of the human figure, with removable organs, which can be handled and studied by each student, tend to make the elements of anatomy and physiology thoroughly interesting and comprehensible, instead of the jargon into which they too often degenerate with uninstructed persons. At the conclusion of each course the assistant surgeon, or physician, who gives the lectures sets examination papers, which are corrected and marked by the senior surgeon or physician. Classes are held by the home sister in connection with these lectures, at which probationers produce their notes and receive instruction.

### Practical Instruction.

We are strongly of opinion that lectures are of little profit at this early stage in the training, unless supplemented in this way by class work to help the backward, and stimulate all to more thorough mastery of the subjects. Practical instruction in general nursing, bed-making, taking of temperatures, etc., is continuously given by the ward sisters to their probationers, and there are classes on bandaging and on instruments. Reports are furnished by each sister every three months on the progress made, and these reports are sent in to the matron.

### The Nurses' Record-Book.

Books are provided in which the nurse has to keep a complete record of her training, and these books, duly "signed up," have to be produced to the committee before the certificate is granted. The system is an excellent one, as it enables the extent of the nurse's instruction, both theoretical and practical, to be grasped at a glance. We append a brief synopsis of the Record-Book.

London Homœopathic Hospital.

Name ..................................................................

Date of Engagement ................................................

*Nurse's Training and Instruction.*

It is the duty of the Nurse to keep this record accurately.

The entries as to the Ward Service must be signed by the Matron.

The entries as to Lectures and Practical Instruction must be signed by the Lecturer or Instructor.

The record is to be placed in the Matron's Office on May 1 in each of the three years of training, and as often as requested by her.

*Ward Training—First Year.*

| Date | Duties | Signatures and Remarks |
|---|---|---|
| | | |

[Similar pages are provided for Second and Third Year.]

*Ward or Private Nursing Work—Fourth Year.*

| Date | Duties | Signatures and Remarks |
|---|---|---|
| | | |

Each Lecture or Class is to be recorded with the date.

Name ..................................................................

Lectures and Practical Instruction.

*First Year.*

| Date | Nature of Lecture or Instruction | Lecturer or Instructor | Signatures and Remarks |
|---|---|---|---|
| | | | |

[Similar pages are provided for the Second Year.]

*Special Lectures, etc.*

| Date | Nature of Lecture or Instruction | Lecturer or Instructor | Signatures and Remarks |
|---|---|---|---|
| | | | |

* Previous articles appeared in The Hospital of May 16 and 30, June 13, July 11 and 25, August 8, 1914, and January 16, 1915.

### Remainder of the Course.

The training proper lasts three years. In the second and third years the lectures are on Nursing Ethics, on Minor Surgery, on Medicine, on Invalid Cookery. Special lectures are also delivered from time to time on Eyes, Ears, Throat, Homœopathy, etc. After each course pupils are tested by examinations. The ward scheme provides for the senior or second year probationers to spend three months alternately on day and night duty. In the third year they are promoted, if suitable, to be staff nurses, and then remain four or six months in their ward. All the nurses receive good training in theatre work for at least three or four months. The general theatre staff comprises sister, staff nurse, and one probationer, usually in her first year, who gets further theatre experience later on in her course. In addition to the ordinary theatre there is one attached to the paying-wards, and the experience available in the paying-wards is of the greatest possible advantage to nurses desirous of taking up private work.

### Examination by Outside Matron.

The final examination has to be taken at the end of the third year. Four years ago the hospital began to appoint an outside examiner for this purpose from among the matrons of the larger London hospitals. A different matron is examiner each year, and this innovation has proved very successful. The papers are both practical and theoretical with viva voce and practical tests, and the examiner sends in a written report to the committee. The Matron highly approves of the system, and considers it has had an excellent effect on all who are engaged in training the probationers. A gold medal is awarded to the nurse who gains the highest number of marks for ward reports and examinations during her training.

### Midwifery and Massage.

These subjects do not form part of the ordinary curriculum. An arrangement has been made, however, through which nurses who have completed their fourth year's service may receive training in massage and midwifery in other institutions on condition of returning to serve the hospital subsequently for an additional year after the conclusion of the training.

### The Private Staff.

After the nurse has completed her training and passed her examination she may be sent out to do private nursing. The private staff numbers from fifteen to twenty. When not at cases the fourth-year nurses return to the wards or to the pay-wards, and take holiday and emergency work as required. After this year they are allowed, should they prefer it, to work at the Nurses' Institute on the co-operative system. No profits beyond the general upkeep of the institute are made on the private nursing staff, and the accounts are kept distinct from those of the hospital.

### The Nurses' Home.

This new building was only opened in 1913, and is a fine addition to the hospital, which it exactly faces in Great Ormond Street. It has been constructed at great expense, and with most sympathetic consideration for the nurses' comfort and convenience. Good separate bedrooms are provided for each member of the staff. The sitting-rooms are quite unusually spacious and numerous, and separate rooms are provided for the sisters, staff nurses, and probationers, besides a writing-room and a fine recreation room in the basement, and a badminton court outside. The dining-hall is next to the kitchen in the hospital building. The rooms are amply supplied with papers and periodicals by supporters of the hospital. There are no sick-rooms in the home, nurses not being kept there more than forty-eight hours if ill, but are warded in one of the paying-wards if available, or in one of the smaller wards of the hospital.

### Pay and Prospects.

There is no premium, and the salaries are £8, £12, £18, £25. Sisters' salaries run from £35 to £40. A very good opening is afforded to nurses suitable for private work by the Nurses' Institute, and a steady demand exists for their services. The nurses trained at the hospital take a good place also in other institutions, being well fitted for home and Colonial appointments, many of which they secure. The warm interest taken by subscribers in all which affects the nurses' interests is a marked feature of the school.

